# Screening lifespan-extending drugs in Caenorhabditis elegans via label propagation on drug-protein networks

**DOI:** 10.1186/s12918-016-0362-4

**Published:** 2016-12-23

**Authors:** Hui Liu, Mengmeng Guo, Ting Xue, Jihong Guan, Libo Luo, Ziheng Zhuang

**Affiliations:** 1 Changzhou University, Jiangsu, 213164 China; 2 Changzhou NO. 7 People’s Hospital, Changzhou, Jiangsu, 213011 China; 3 Department of Computer Science and Technology, Tongji University, Shanghai, 201804 China

**Keywords:** Longevity, Label propagation, Drug targets, Caenorhabditis elegans

## Abstract

**Background:**

One of the most challenging tasks in the exploration of anti-aging is to discover drugs that can promote longevity and delay the incidence of age-associated diseases of human. Up to date, a number of drugs, including some antioxidants, metabolites and synthetic compounds, have been found to effectively delay the aging of nematodes and insects.

**Results:**

We proposed a label propagation algorithm on drug-protein network to infer drugs that can extend the lifespan of C. elegans. We collected a set of drugs of which functions on lifespan extension of C. elegans have been reliably determined, and then built a large-scale drug-protein network by collecting a set of high-confidence drugprotein interactions. A label propagation algorithm was run on the drug-protein bipartite network to predict new drugs with lifespan-extending effect on C. elegans. We calibrated the performance of the proposed method by conducting performance comparison with two classical models, kNN and SVM. We also showed that the screened drugs significantly mediate in the aging-related pathways, and have higher chemical similarities to the effective drugs than ineffective drugs in promoting longevity of C. elegans. Moreover, we carried out wet-lab experiments to verify a screened drugs, 2- Bromo-4’-nitroacetophenone, and found that it can effectively extend the lifespan of C. elegans. These results showed that our method is effective in screening lifespanextending drugs in C. elegans.

**Conclusions:**

In this paper, we proposed a semi-supervised algorithm to predict drugs with lifespan-extending effects on C. elegans. *In silico* empirical evaluations and in vivo experiments in C. elegans have demonstrated that our method can effectively narrow down the scope of candidate drugs needed to be verified by wet lab experiments.

**Electronic supplementary material:**

The online version of this article (doi:10.1186/s12918-016-0362-4) contains supplementary material, which is available to authorized users.

## Background

Aging is a physiological process in accompany with continuous accumulation of damages to cells and organs, which gradually lead to loss of normal organ functions and rise vulnerability to disease [1]. With the increasing average age of people, the risk of age-related diseases also concomitantly increases. Therefore, there is a rising interest in exploring drugs to promote healthy longevity, which basically aim at preventing or delaying the onset of age-associated illnesses, such as cardiovascular disease, type 2 diabetes, neurodegenerative disease and cancer [2, 3].

It has been shown that restriction of energy intake can effectively extend lifespan of diverse species from yeast and worms to mammals [4, 5]. Dietary restriction can also protect against age-related risk factors of diabetes, cardiovascular disease, and cancer in human [6]. Previous studies have verified that the beneficial effects of dietary restriction in mammals are primarily obtained by increasing insulin sensitivity and decreasing blood glucose in mammals [7, 8]. Also, reduced activity of nutrient-sensing pathways, including IIS (insulin/insulin-like growth factor), AMPK and mTOR signaling pathways, can slow down the aging of yeast, worms, flies and mice [9–12], suggesting that inhibition of these signaling pathways may induce a physiological state similar to that resulting from food shortage. Moreover, the lifespan-control mechanisms are remarkably conserved across diverse species [13].

Up to date, a number of drugs, including some antioxidants, metabolites and synthetic compounds, have been found to effectively delay the aging of nematodes and insects [14–16]. Some natural products and extract from plants can also extend the lifespan of invertebrates [17–20]. In particular, some drugs approved for human disease can promote the longevity of worm and mice, including Rapamycin [14], Aspirin [21], Metformin [22] and Resveratrol [23]. These findings imply there exist some common physiological mechanism between aging and the diseases treated by these drugs [24].

Some public data resources have been released for exploring aging [25–27]. For instance, GenAge, the benchmark database of genes related to ageing, has collected the genes associated with changes in the ageing phenotype or longevity in human and four model organisms [26]. All gene entries in GenAge are compiled from experimentally validated results published in peer-reviewed scientific literature. A gene is considered for inclusion if genetic manipulations (including knockout, mutations, overexpression or RNA interference) result in noticeable changes in the ageing phenotype and/or lifespan. NetAge is another aging-related web resource, which provides access to gene, protein and miRNA interaction networks that are involved in complex processes of aging and age-related diseases [27]. Gene Aging Nexus (GAN) have collected together numbers of ageing-related microarray gene expression data from human, rat, mouse, fruitfly, worm and yeast studies [28]. These public databases provide a valuable resource for us to develop computational methods for screening lifespan-extending drugs.

It is worth noting that the model organism Caenorhabditis elegans (*C. elegans*) has advantageous features for aging exploration, including its short lifespan, stereotypical development and small size [29]. These features make it a popular model species to conduct whole-organism assessment of anti-aging effect and mechanism of action of drugs [30, 31]. Thanks to the evolutionarily conserved mechanism of lifespan control from worm to mice and human, potential anti-aging drugs can be tested on worms and then transferred to mammals. On the other hand, drug efficacy is primarily exerted through inhibiting (or activating) the functions of target proteins by drug molecules, which specifically bind to the protein functional domains so that the corresponding biological functions are desirably blocked (or catalyzed) [32]. As a result, drug-protein interactions have been intensively studied and exploited in the drug research and development [33–35].

In this paper, we proposed a semi-supervised learning algorithm to predict drugs with lifespan-extending effects on *C.elegans*. We built a set of drugs and genes of which (effective or ineffective) influence on lifespan of C. elegans has been experimentally determined. The drug set included 1,309 drugs, which were collected from a large-scale bioassay screening for anti-aging drugs [36], and manually curated anti-aging drugs from literature [31]. The gene set included 681 genes, which were collected from the aging-related benchmark database GenAge [26]. By extracting a set of high-confidence drug-protein interactions from STITCH [37], we built a drug-protein bipartite network and then run a label propagation algorithm to predict new effective drugs. To calibrate the performance of the proposed method, we conducted 5-fold cross-validations on the gold-standard set of drugs. The empirical experiments showed that our method achieves higher performance than two classical models, kNN and SVM. Moreover, our screened drugs significantly mediate in the aging-related pathways, and have higher chemical similarities to the effective drugs than ineffective drugs in promoting longevity in C. elegans. Finally, we carried out wet-lab experiments to test the effectiveness of one screened drugs, 2-Bromo-4’-nitroacetophenone (PubChem CID000066840), and found that it can significantly promote the longevity of *C.elegans*. Both the *in silico* and in vivo experimental results demonstrated the performance of our computational approach for screening anti-aging drugs.

## Results

### Performance evaluation by 5-fold cross-validations

**Fig. 1:**
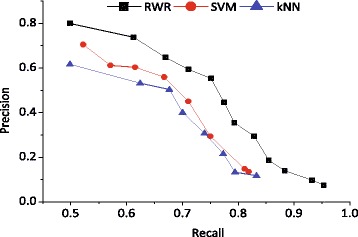
The recall-precision curves of our random walk with restart on drug-target network, as well as the two classical classifiers kNN and SVM. The recall and precision measures were computed with respect to the value of threshold *θ*

We conducted 5-fold cross-validations on the gold-standard set of drugs (see Data resources for details about the dataset) to evaluate the performance of our method. The drugs in the gold standard set were randomly split into 5 subsets with roughly equal size, and then each subset was taken in turn as a test set and the remaining four subsets were taken as input to run our method. The prediction accuracy was evaluated on the test set, and the averages over the 5-fold test subsets are regarded as final performance measures. Based on the predicted scores by our method and two classical classifiers, kNN and SVM, we computed the precision and recall measures for a given threshold, i.e. the drugs with scores greater than the threshold were classified as positive (effective) ones, and negative (ineffective) ones otherwise. We built the recall-precision curves for the three methods by gradually increasing the threshold, as shown in Fig. 1. It can be found that our method significantly outperformed the two classical classifiers kNN and SVM.

### Highly scored drugs mediate in aging-related pathways

**Fig. 2:**
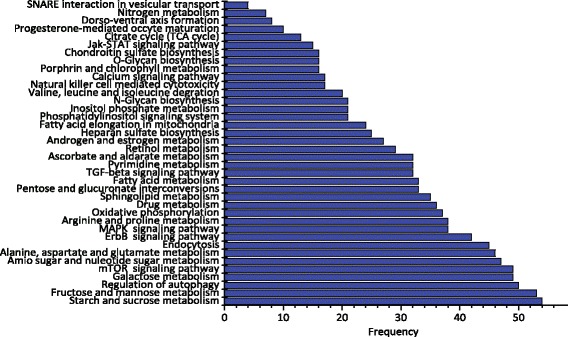
The frequency distribution signaling and metabolism pathways in which the target proteins of top 100 screened drugs are significantly enriched. The pathway enrichment analysis was conducted using DAVID V6.7 [38]

We carried out pathway analysis based on the target proteins of 100 top-rank drugs predicted by our method (for whole list of the predicted drugs see Additional file 1). The target proteins of each drug are obtained from STITCH database, and the pathway analysis were conducted by using DAVID V6.7 [38]. We found that screened drugs primarily mediate in sugar catabolism, energy metabolism and other processes related to cellular detoxification, as shown in Fig. 2. The results are consistent with several previous studies that have shown that these pathways mediate in the aging processes [39–41]. For example, McElwee et al. analyzed the differentially expressed genes during the aging processes of mice, fly and worm, and identified a group of evolutionarily conserved biological processes related to aging, including sugar and energy catabolism [40]. Moreover, de Magalhaes et al. performed a meta-analysis of publicly available age-related expression microarray datasets on healthy and non-treated adult specimens, and then identified consistently under- or over-expressed genes related to aging processes. The enrichment analysis based on this set of aging-related genes demonstrated that various pathways mediate in aging processes across species, including inflammation and immune response, energy metabolism, etc [41]. The pathway enrichment analysis results of our screened drugs are notably consistent with the conclusions of previous studies, which indeed support the top-rank drugs are potential anti-aging agents.

**Fig. 3:**
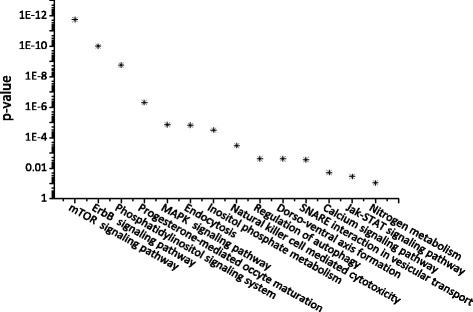
The signaling and metabolism pathways analysis based on the 309 target proteins of the drug with highest *F*-ratio score, ZINC218147572 (PubChem CID100005691). The pathway enrichment analysis was conducted using DAVID V6.7 [38]

**Fig. 4:**
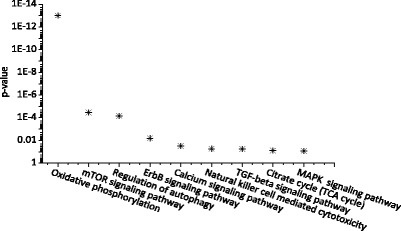
The signaling and metabolism pathways analysis based on the 681 aging-related genes in GenAge database. The pathway enrichment analysis was conducted using DAVID V6.7 [38]

In particular, we found that the drug with the highest F-ratio score, ZINC218147572 (PubChem CID100005691), has 309 target proteins deposited in STITCH. The pathway analysis by DAVID showed that this drug significantly mediates in TOR signaling pathway (*p*-value=1.8e-12), as shown in Fig. 3. It has been confirmed that inhibition of TOR pathway can slow down aging and extend healthy lifespan in diverse organisms, including yeast, worms, flies and mice [1, 42]. Interestingly, we conducted pathway enrichment analysis by using the 681 genes in GenAge, as shown in Fig. 4. It can be seen that there are remarkable overlaps between the significant pathways in which the two different set of genes are enriched. (for screenshots of the pathway analysis results see Additional file 2). We strongly suggest that ZINC218147572 is a promising drug for lifespan extension. We would like to conduct wet-lab experiments to test its effectiveness on lifespan extension of *C.elegans*, once we get the drug in future (we failed to buy or produce this drug during the preparation of this paper).

### Screened drugs have higher chemical similarity to effective drugs than ineffective drugs

**Fig. 5:**
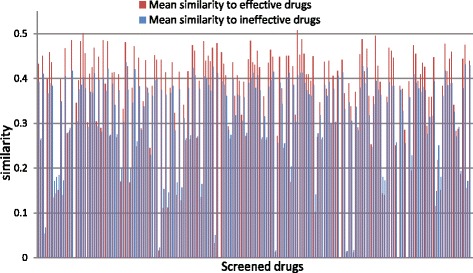
The mean chemical similarity of the 195 screened drugs (*F*-ratio ≥2) to the effective and ineffective drugs included in the training set. The chemical similarities were calculated according to Eq. (6), on the basis of chemical attributes retrieved from PubChem [43]

We also checked whether the screened drugs have higher chemical similarity to the known effective drugs than ineffective drugs in promoting lifespan or not. For this purpose, we selected 195 drugs of which the *F*-ratio are greater than 2. Based on the chemical fingerprints obtained from PubChem [43], we compute the chemical similarities between the 195 screened drugs and the set of known effective/ineffective drugs. The similarity measure is defined as the cosine angle of the chemical attribute vectors of two drugs, as describe in Eq. (6). For convenience of presentation, we computed the mean similarity to the effective/ineffective drugs for each screened drug. As shown in Fig. 5, tt can be found that the screened drugs have significantly higher mean similarities to the effective drugs than the ineffective drugs. Furthermore, we conducted a pair-sample *t*-test against the null hypothesis that the mean similarity to effective drugs is not greater than that to ineffective drugs (degree of freedom, *df*=194). The result of pair-sample *t*-test indicated that the null hypothesis should be rejected and accept alternative hypothesis (*p*-value ≤2.33×10^−24^).

### Wet-lab validation of effectiveness of one screened drug

**Fig. 6:**
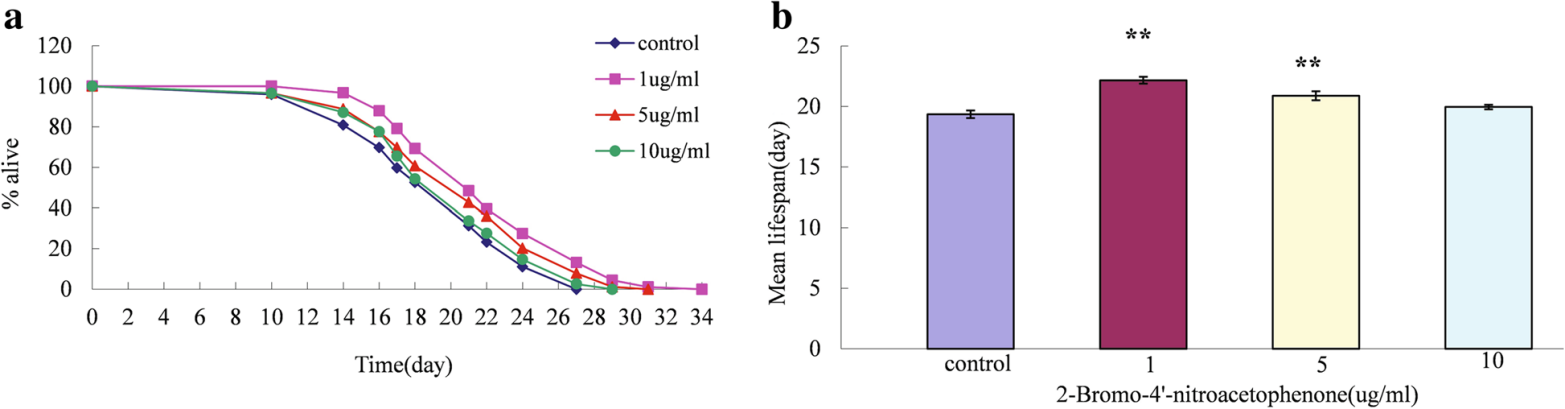
The screened drug, 2-Bromo-4’-nitroacetophenone (PubChem CID000066840), effectively extends the lifespan of *C.elegans*. **a** Survival curves of the animals under control and treated by three different concentration, 1ug/ml, 5 ug/ml and 10 ug/ml, respectively. **b** Bar charts of the mean lifespans in nematodes. (**P-value <0.01)

To further validate the performance of our method, we have conducted wet-lab experiments to check the effectiveness of one screened drug, 2-Bromo-4’-nitroacetophenone (PubChem CID000066840), on the lifespan extension of *C. elegans*. Nematodes were treated with 2-Bromo-4’-nitroacetophenone at three different concentrations, 1 ug/ml, 5 ug/ml and 10 ug/ml, during their lifespan in order to study the effect of 2-Bromo-4’-nitroacetophenone on lifespan of C.elegans. As shown in Fig. 6 a and b, the drug showed concentration-dependent effects on the lifespan extension of C. elegans. The nematodes treated with the drug at the concentration of 1ug/ml and 5 ug/ml showed significantly extended lifespan compared with untreated animals (one-way AVONA test, *p*-value <0.01). The results of the wet-lab experiments provided strong support for the performance of our method.

## Data resources

### Drugs

The Library of Pharmacologically Active Compounds (LOPAC^1280^) contains 1,280 types of compounds that can be categorized into 55 pharmacological classes according to their mammalian targets. A recent high-throughput biochemical assay has been conducted to screen compounds in LOPAC^1280^ to identify drugs that can increase *C. elegans* lifespan [36]. As a result, 60 compounds in LOPAC^1280^ have been identified as effective drugs in promoting longevity of *C. elegans*, and the remaining drugs are ineffective drugs. Besides, Lucanic et al. have manually curated 29 types of synthetic compounds and natural products that can promote longevity of *C. elegans* from literature in their review paper [31]. Based on the known effective or ineffective drugs, we built a set of drugs including 1,312 compounds and natural products in total. In particular, 89 effective drugs (60 compounds in LOPAC^1280^ plus 29 manually curated synthetic compounds and natural products) were labeled as positive samples, and the remaining 1,220 drugs in LOPAC^1280^ were labeled as negative samples (detailed information of the drugs is presented in Additional file 3).

To build the test set of drugs, we extracted 1,991 FDA-approved small molecule drugs and 207 FDA-approved biotech (protein/peptide) drugs from DrugBank database (V5.0). Totally, 2,198 FDA-approved drugs were included in the test set.

### Aging-related genes

Aging-related genes were obtained from GenAge, which is the benchmark database of aging-related genes published by Human Ageing Genomic Resources (HAGR) [26]. GenAge covers the aging-related genes in human and four model organisms, including *Mus musculus*, *Drosophila melanogaster*, worm *Caenorhabditis elegans*, and yeast *Saccharomyces cerevisiae*. Each entry in GenAge is a manually curated by experts to ensure high-quality content. We obtained 681 gene entries for *Caenorhabditis elegans* from GenAge. As a gene may encode multiple different proteins due to the alternative splicing that is prevalent in eukaryote, there are 1,481 unique proteins encoded by these aging-related genes (The aging-related genes and the corresponding proteins are presented in Additional file 4).

### Drug-protein bipartite network

Drug-protein interactions were downloaded from STITCH 4.0 [37]. STITCH is a comprehensive database that collects drug-protein interactions from four different sources: experiments, databases, text mining and predicted interactions. Meanwhile, STITCH has calculated an integrative confidence score for each drug-protein interaction, which indicates the confidence of the interaction supported by four types of evidence, i.e. experimental validation, manually curated databases, text mining and predicted interactions. To guarantee high confidence of the drug-protein interactions, we selected only the drug-protein interactions with experimentally supportive or external database evidences.

Besides, we found that most drugs in LOPAC ^1280^ have numbers of target proteins in human, but relatively less target proteins in C. elegans, through retrieval of STITCH. Considering that the scarcity of proteins associated to the effective and ineffective drugs will lead to a small number of training samples in the drug-protein network, we search for orthologous proteins in *C. elegans* of the known target proteins in human. We exploited OrthoList, a compendium of orthologous genes/proteins between *C. elegans* and human, to map the human proteins to orthologs in *C. elegans*. Orthologous genes in OrthoList are compiled from four orthology prediction methods, including InParanoid [44], OrthoMCL [45], HomoloGene and Ensembl Compara [46]. To ensure the quality of the orthologous proteins, we selected only the orthologous proteins if the orthologous proteins have been predicted by at least two methods. As a result, we got 2,518,944 high-confidence interactions between 397,258 drugs and 9,249 proteins in *C. elegans*.

**Fig. 7:**
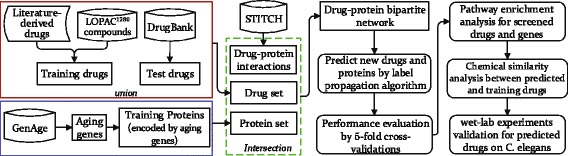
The flowchart for illustrating the data resources and procedures of the proposed methods. It clearly describes the construction of the drug-protein bipartite network from various data resources, prediction of new anti-aging drugs via label propagation, as well as downstream evaluation and analysis procedures including cross-validation, gene set enrichment analysis and wet-lab experiment validations

The drug-protein bipartite network was constructed by choosing the high-confidence interactions of which drugs or proteins were included in the drug set or aging-related gene set mentioned above. After removal of small disconnected subnetworks, we got a drug-protein bipartite network with 18,317 drug-protein interactions between 813 unique drugs and 3,660 unique proteins (Detailed information is presented in Additional file 5). In particular, there are 450 known effective/ineffective drugs (34 effective drugs and 416 ineffective drugs) and 20 known aging-related genes among the drug-protein bipartite network, which is used as gold-standard set in the following empirical experiments to evaluate the performance of our method. The data resources and construction of the drug-protein network are illustrated in Fig. 7.

## Methods

### Random walk with restart on drug-protein network

**Fig. 8:**
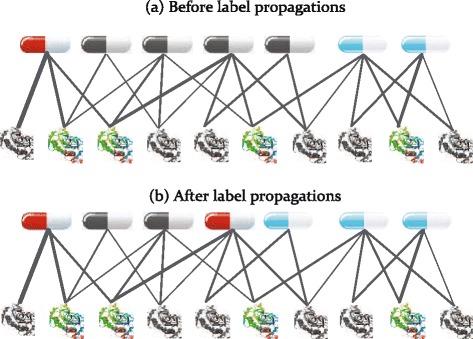
The illustrative diagram of label propagation algorithm run on the drug-protein bipartite network. The *red and blue nodes* represent the known effective and ineffective drug nodes, from which the random walker begin the random walk process

We consider the problem of predict new lifespan-extending drugs in terms of label propagation on the drug-protein bipartite network. Our inspiration is that a small number of experimentally effective and ineffective drugs are labeled as positive and negative samples, and their labels might be propagated to other nodes along the edges of the drug-protein network. Figure 8 illustrates the label propagation starting from a few initially labeled nodes. The effective and ineffective drugs are respectively shown in red color and cyan color, and candidate drugs are shown in gray. The weight of the edges are proportional to the confidence scores of the drug-protein interactions.

Based on the drug-protein network, we start two independent random walks with restart from the effective and ineffective drugs, respectively. After convergence, we obtain a stationary distribution specifying the probability that a random walk with restart will arrive at each node. The drugs that can be reached with higher probability from effective drugs than from ineffective drugs are classified as effective ones, or ineffective ones otherwise.

Formally, suppose we have *m* drugs and *n* proteins of interest, and a set of drug-protein interactions with quantitative confidence scores. Denote by *V*_*d*_ and *V*_*t*_ the sets of drug and protein nodes, and *E*={*E*(*i,j*)} a set of edges connecting drugs and proteins, in which *i* belongs to *V*_*d*_ (*i*=1..*m*) and *j* belongs to *V*_*t*_ (*j*=1..*n*). Let *V*=*V*_*d*_∪*V*_*t*_, we construct an undirected network *G*=<*V, E*>. We define a symmetric affinity matrix *A*, where *A*(*i,j*) is equal to the confidence score of the corresponding interaction if *E*(*i,j*)∈*E*, or 0 otherwise. Define *D* as the diagonal matrix of degrees of the nodes, i.e. $D(i,i)=\sum _{j} A(i,j)$, we constructed the transition matrix *T* as below:

1 $$ T=D^{-1}A  $$

To conduct two independent random walks, we introduce an initial label matrix *L* with |*V*| rows and 2 columns, where the two column vectors are indexed according to the effectiveness of drugs that have been experimentally verified. The values of matrix *L* are given by: 1) *L*(*i*,1)=1 if drug *i* is ineffective in promoting longevity; 2) *L*(*i*,2)=1 if drug *i* is effective in promoting longevity; and 3) 0 for all other elements. Note that our method can simultaneously predict lifespan-extending drugs and aging-related genes, by setting the initial label matrix *L* to include both experimentally validated drugs and known aging-related genes, i.e. the element of second column *L*(*i*,2)=1 if the *i*-th node is effective drug or aging-related gene coding proteins.

Before starting the random walk, each column of the initial label matrix *L* was normalized to be a probability distribution:

2 $$ L'=(D_{L}^{-1}L^{T})^{T},  $$

in which *D*_*L*_ is a diagonal matrix with $D_{L}(i,i)=\sum _{j}L(j,i)$. We allow the restart of the walk in every step at a source node with probability *α*, the random walk process can be formulated as:

3 $$ P(t+1)=(1-\alpha)TP(t)+\alpha L',  $$

where *P*(*t*) is a |*V*|×2 matrix and *P*(0)=*L* ^′^.

Let *P* ^∗^ be the matrix when the random walks converge, i.e. the change between *P*(*t*) and *P*(*t*+1) (measured by the L1 norm) is less than very small number *ε*. The two columns of *P* ^∗^ are the two stationary distributions that specify the probability to reach each node, corresponding to the two separately random walks with restart from the effective and ineffective drug nodes, respectively. Once we obtained *P* ^∗^, we computed the odds ratio, called *F*-ratio as *F*(*i*)=*P* ^∗^(*i*,2)/*P* ^∗^(*i*,1) for each candidate drug *i*. If *F*(*i*) is greater than a predefined threshold, drug *i* is classified as effective one in promoting longevity. To build recall-precision curve, we ranked all candidate drugs according to their *F*-ratio values and gradually increase the value of threshold *θ* used for classification decision. In Algorithm 1, we outline the steps of the label propagation algorithm on the drug-protein network. Moreover, an illustrative flowchart for our method is presented in Fig. 7.



### Drug similarity

For each drug, we obtained the SMILES from PubChem database [43] and then generated the chemical features and fingerprints using PaDEL software (release v2.21) [47]. PaDEL takes as input the SMILES of each drug to calculate 1D and 2D physicochemical descriptive features and fingerprints. For each drug, the attributes consist of 1,445 descriptive features, including such as atom count, bond count, molecular weight, xlogP, and 880 chemical fingerprints. Subsequently, we removed the features with the same attribute values across all drugs, and obtained 1,744 chemical attributes for each compound.

Based on the chemical attributes, we computed the chemical similarity between each pair of drugs. Formally, each drug was represented by a 1,744-dimension vector, in which the element is equal to the value outputted by PaDEL, or 0 otherwise. Denote by $\vec {c}_{i}=\{c_{i1},c_{i2},\ldots,c_{iK}\}$ and $\vec {c}_{j}=\{c_{j1},c_{j2},\ldots,c_{jK}\}$ the chemical attribute vectors of drugs *i* and drug *j*, we computed the *cosine similarity* as below:

4 $$  S_{d}^{(1)}(i,j) = \frac{\sum_{k=1}^{K} c_{ik}c_{jk}}{\sqrt{\sum_{k=1}^{K} c_{ik}^{2}}\sqrt{\sum_{k=1}^{K} c_{jk}^{2}}},  $$

in which *K* is the length of the chemical attribute vector (*K*=1774).

Also, we computed the drug-drug similarities, referred to as *target sharing similarity* based on our built drug-protein interaction network. Each drug was represented by a 3,660-dimension binary vector whose element is 1 if the corresponding protein is targeted by the drug and otherwise 0. We built the target sharing similarity matrix based on the number of shared targets between two drugs. This similarity measure is defined as the ratio of number of common target proteins to the total number of target proteins of the two drugs. This measure is actually equivalent to *Jaccard score* that can be mathematically defined as:

5 $$ S_{d}^{(2)}(i,j)=\frac{|t_{i}\cap t_{j}|}{|t_{i}\cup t_{j}|}  $$

in which *t*_*i*_ and *t*_*j*_ are the binary vectors representing the proteins targeted by drug *i* and drug *j*, respectively.

Since we obtained two similarity measures derived from different attributes for each pair of drugs, we thus integrated the two similarity measures into a comprehensive similarity measure as below:

6 $$  S_{d}(ij) = 1-\prod_{n}(1-S^{(n)}_{d}(ij)),  $$

in which $S^{(1)}_{d}(ij)$ and $S^{(2)}_{d}(ij)$ represent the similarity measures between *i* and *j*-th drugs derived from chemical attributes and target proteins, respectively.

### Prediction using classical classifiers

To calibrate the performance of our proposed method, two classical classifiers, k-Nearest Neighbor(kNN) and Support Vector Machine (SVM), were used to predict the lifespan-extending drugs. The k-Nearest Neighbor(kNN) was run by using Weka 3.7 [48], and SVM was run by using libsvm 3.17 [49]. To apply the kNN classifier, we convert the the similarity measure defined in Eq. (6) to the distance measure by computing 1−*S*_*d*_ *ij*. The parameter *k* was set to 1, 3 and 5, respectively. As similar results were obtained for different *k* values, we reported only the results of *k*=3. For SVM, We concatenated the chemical attributes and the target proteins of each drug, and thus obtain a 5,434-dimension vector that is taken as input by libsvm. The radial basis function kernel was used, and other parameters were tuned by 10-fold cross validations. The experimentally verified drugs are labeled as positive and negative sample and used to train the SVM classifier, and the learned model was then used to classify the set of test drugs.

### Reagents and worm strain maintenance

2-Bromo-4’-nitroacetophenone (PubChem CID000066840) was obtained from Sigma-Aldrich (St. Louis, MO, USA). Wild-type C. elegans N2, which is originally obtained from Caenorhabditis Genetics Center, was maintained on nematode growth medium (NGM) plates seeded with Escherichia coli OP50 at 20C as described previously [50]. Age synchronous populations of nematodes were obtained as described [51]. The compound was added to the NGM plates just before plating.

### Lifespan assay

The lifespan assay was performed basically as described in [52]. 2-Bromo-4’-nitroacetophenone treatment was performed throughout the lifespan from the stage of L4-larvae. The worms were transferred daily for the first several days of adulthood during the assay. The nematodes were checked at intervals during the lifespan when they began to die and worms were considered as dead when they did not respond to the stimulation of a platinum wire.

## Discussions and conclusions

It is of frequent occurrence that labeled samples, which mean their functions were experimentally validated, are extremely rare and unlabeled data is abundant. Therefore, many semi-supervised algorithms have been developed, including self-training, S3VMs and graph-based methods, which can fully utilize fewer labeled data and large unlabeled data in the learning and predicting processes. The merit of network-based semi-supervised algorithms lie in that they can take full advantage of the inherent structure between labeled and unlabeled data. In this paper, we employed the random walk with restart on drug-protein bipartite network to predict drugs with lifespan-extending effects on *C.elegans*. In fact, label propagation on bipartite networks has been widely used in various fields, such as drug repositioning [53], personal recommendation [54] and political polarity classification [55].

To the best of our knowledge, we are the first to propose the computational method for predicting lifespan-extending drugs in term of drug-protein interactions. To calibrate the performance of the proposed method, we employed two classical models, kNN and SVM, in the empirical evaluation of the performance, as we have not found any other existing computational methods for screening lifespan-extending drugs in term of drug-protein interactions. The 5-fold cross-validations on the gold-standard dataset showed that our method achieve higher performance than the two classical classifiers.

Our wet-lab experiments verified that one of the screened drugs, 2-Bromo-4’-nitroacetophenone (PubChem CID000066840), can significantly promote the longevity of *C.elegans*. In our future work, we will carry out systematic wet-lab experiments to verify what percentage of the screened drugs are effective in promoting longevity of C. elegans, and explore the biological mechanism of anti-aging effect of the drugs in pathway and gene levels.

In summary, we proposed a semi-supervised classification algorithm, random walk with restart on bipartite graph, to predict drugs with lifespan-extending effects on C.elegans. Our method aims at predicting lifespan-extending drugs in a large scale, and narrowing down the scope of candidate drugs needed to be verified by wet-lab experiments. The results of empirical experiments and wet-lab experiment show that our computational screening approach achieves state-of-the-art performance.

## Additional files

Additional file 1 Detailed information of the predicted drugs, including the probabilities derived from two random walks independently started from effective and ineffective drugs, as well as the *F*-ratio. (XLSX 281 kb)

Additional file 2 The screenshots of the pathway enrichment analysis results based on the 309 target proteins of drug ZINC218147572 and the set of 681 aging-related genes in GenAge, respectively. (DOCX 383 kb)

Additional file 3 Detailed information of the drugs collected from bioassay screening result of the LOPAC ^1280^, as well as the manually curated anti-aging drugs from literature review. (XLSX 180 kb)

Additional file 4 The aging-related genes of *C. elegans* extracted from GenAge database, together with the corresponding transcripts of each aging-related genes. (XLSX 99 kb)

Additional file 5 The drug-disease bipartite network used in the paper, which includes 18,317 drug-protein interactions between 813 unique drugs and 3,660 unique proteins. (XLSX 440 kb)
