# Supplementary material for: Screening lifespan-extending drugs in Caenorhabditis elegans via label propagation on drug-protein networks
Source: BMC Syst Biol. 2016 Dec 23;10(Suppl 4):131. doi: 10.1186/s12918-016-0362-4 (PMC5260106; doi:10.1186/s12918-016-0362-4)
Supplement: Additional file 2 — The screenshots of the pathway enrichment analysis results based on the 309 target proteins of drug ZINC218147572 and the set of 681 aging-related genes in GenAge, respectively. (DOCX 383 kb) [file 12918_2016_362_MOESM2_ESM.docx]

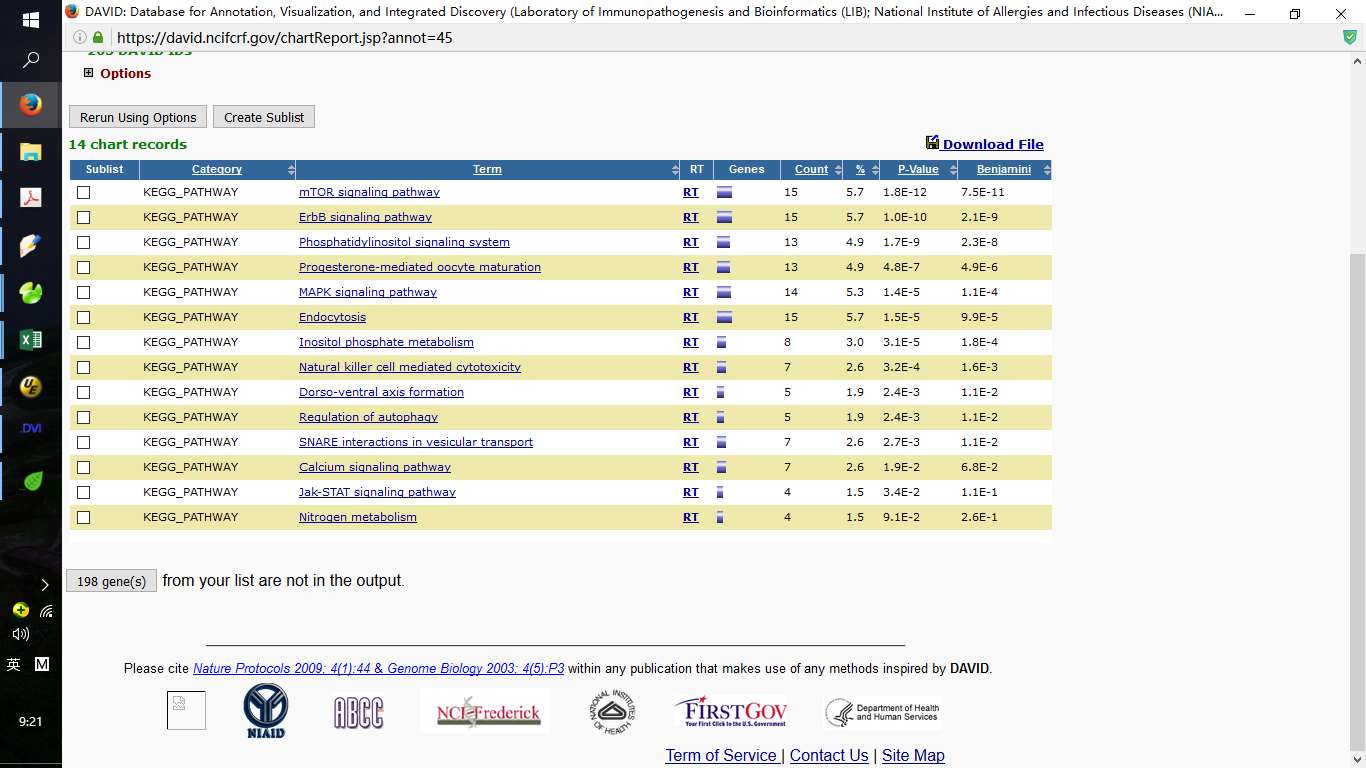


Figure S1. The pathway enrichment analysis results by using the set of 309 target proteins of drug ZINC218147572 (PubChem CID100005691). The analysis tool is DAVID v6.7 release.


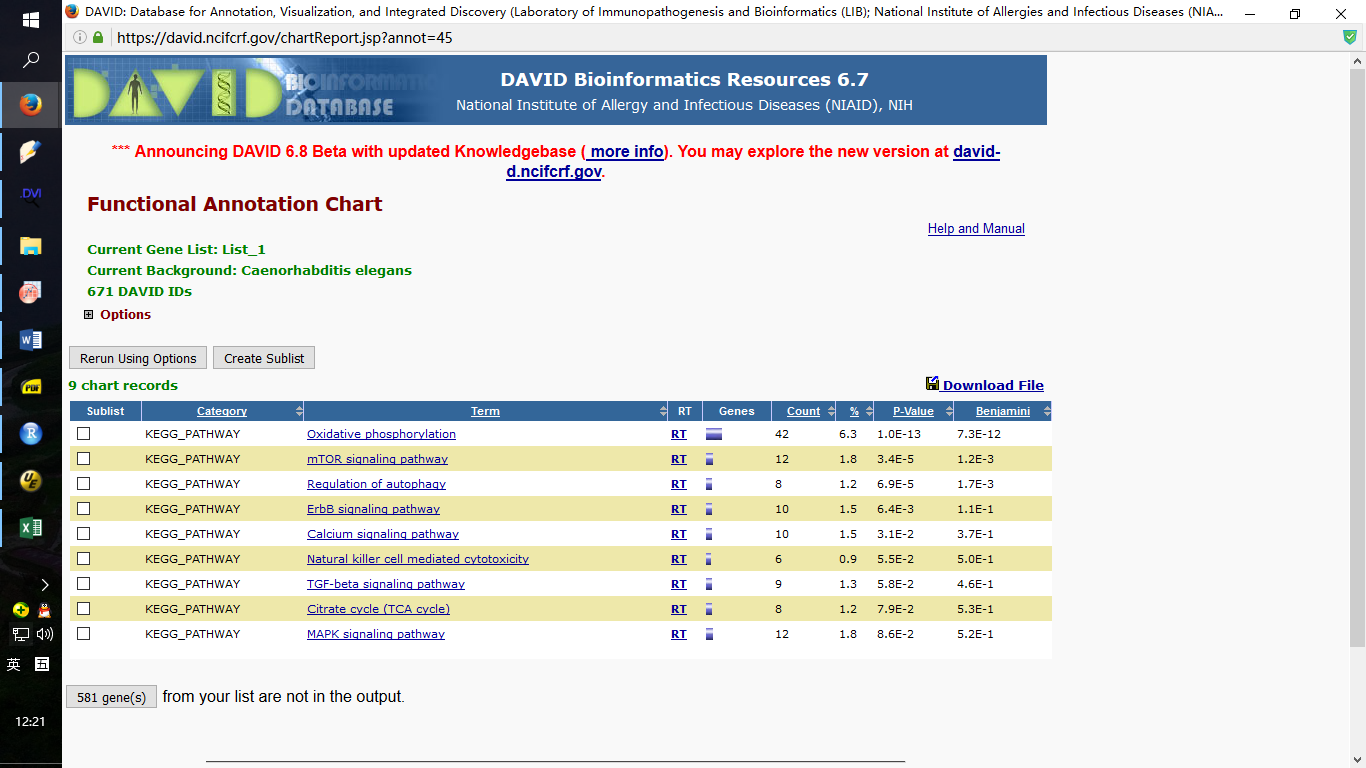


Figure S2. The pathway enrichment analysis results by using the set of 681 aging-related genes in GenAge. The analysis tool is DAVID v6.7 release.
